# Temperature Sensing Properties of Biocompatible Yb/Er-Doped GdF_3_ and YF_3_ Mesocrystals

**DOI:** 10.3390/jfb15010006

**Published:** 2023-12-22

**Authors:** Ivana Dinić, Marina Vuković, Maria Eugenia Rabanal, Milica Milošević, Marta Bukumira, Nina Tomić, Miloš Tomić, Lidija Mančić, Nenad Ignjatović

**Affiliations:** 1 Institute of Technical Science of SASA, 110000 Belgrade, Serbia; ivana.dinic@itn.sanu.ac.rs (I.D.); nina.tomic@itn.sanu.ac.rs (N.T.); milos.tomic@itn.sanu.ac.rs (M.T.); 2 Innovative Centre, Faculty of Chemistry, University of Belgrade, 110000 Belgrade, Serbia; marinav@chem.bg.ac.rs; 3 Department of Materials Science and Engineering and Chemical Engineering, Universidad Carlos III de Madrid and IAAB, 28903 Madrid, Spain; eugenia@ing.uc3m.es; 4 Department of Radiation Chemistry and Physics, Vinča Institute of Nuclear Sciences—National Institute of the Republic of Serbia, University of Belgrade, Vinča, 110000 Belgrade, Serbia; milicam@vin.bg.ac.rs; 5 Institute of Physics Belgrade, National Institute of the Republic of Serbia, University of Belgrade, 110000 Belgrade, Serbia; marta@ipb.ac.rs

**Keywords:** optical thermometry, up-conversion, YF_3_, GdF_3_, chitosan, mesocrystals, cytotoxicity

## Abstract

Y_0.8−x_Gd_x_F_3_:Yb/Er mesocrystals with a biocompatible surface and diverse morphological characteristics were successfully synthesized using chitosan-assisted solvothermal processing. Their structural properties, studied using X-ray powder diffraction, Fourier transform infrared spectroscopy, scanning and transmission electron microscopy and energy dispersive X-ray analysis, were further correlated with the up-conversion emission (λ_exc_ = 976 nm) recorded in function of temperature. Based on the change in the visible green emissions originating from the thermally coupled ^2^H_11/2_ and ^4^S_3/2_ levels of Er^3+^, the corresponding LIR was acquired in the physiologically relevant range of temperatures (25–50 °C). The detected absolute sensitivity of about 0.0042 °C^−1^, along with the low cytotoxicity toward both normal human lung fibroblasts (MRC-5) and cancerous lung epithelial (A549) cells, indicate a potential for use in temperature sensing in biomedicine. Additionally, their enhanced internalization in cells, without suppression of cell viability, enabled in vitro labeling of cancer and healthy cells upon 976 nm laser irradiation.

## 1. Introduction

Lanthanide doped up-conversion nanoparticles (Ln-UCNPs) belong to the photoluminescence materials whose optical activity, known as the anti-Stokes emission, is described as a nonlinear optical process of the successive absorption of two or more photons, and the emission of light with a shorter wavelength than the excitation one [1]. Their UC luminescence, superior to that of the transition metal ions doped counterparts, is based on the unique electronic configuration of lanthanide ions, 4f^n^5s^2^5p^6^6s^2^ (*n* = 0–14). All lanthanides, with the exception of ytterbium and cerium, have abundant ladder-like 4f energy levels, with electrons having a low electron-phonon coupling strength due to the shielding from the outer-lying 5s^2^ and 5p^6^ levels. With the removal of 6s^2^ electrons, 4f electrons become optically active, either through 4f or 4f-5d transitions [2]. Among lanthanides, Er^3+^, Ho^3+^ and Tm^3+^ are considered to be the most effective activators [3], while Yb^3+^ is recognized as an ideal sensitizer, due to its simple energy level and a relatively high cross-section for absorption at 980 nm. When co-doped together in different host materials with a low phonon energy (~350 cm^−^^1^), broad band gap (>10 eV) and high chemical stability, these emit intense blue, green or red light under near-infrared (NIR) excitation. At present, the Ln-UCNPs emission covers the whole visible spectrum. Accordingly, Ln-UCNPs have potential application in biomedicine for cell labeling or as theranostic agents, in forensics for latent fingerprint visualization, and in security applications for anti-counterfeiting [3,4,5]. Their innovative use in photovoltaic, agriculture and thermal sensing in medicine has recently been in the focus of scientific research [6,7,8]. In particular, temperature sensing, which relies on change in the luminescence intensity ratio (LIR) of thermally coupled energy levels of lanthanide ions, is highly sensitive, self-referenced and does not require additional calibration. LIR-based thermometers are negligibly dependent on the extrinsic factors and could be used for non-contact temperature measurements in diverse areas and environments. To date, many different Ln-UCNPs are explored for this purpose. In addition to the most efficient hexagonal NaYF_4_:Yb/Er phase, Ln-doped NaGdF_4_, YF_3_, Y_2_O_3_, Y_6_O_5_F_8_, Ba_3_Gd_2_F_12_ and Y_3_Al_5_O_12_ are reported to have excellent temperature sensing capability, even when embedded in glass [9,10,11,12,13,14]. In order to be useful for in vivo temperature sensing and photothermal therapy, Ln-UCNPs need to have a hydrophilic, biocompatible surface, and need to be sensitive in the physiologically relevant range of temperatures. The required surface features are usually ensured through subsequent coating with SiO_2_ or a biocompatible polymeric shell [15]. Another effective approach is in situ capping of UCNPs with amino or carboxylic functional groups during synthesis [16]. As we showed in our previous work, chitosan and poly lactic-co-glycolic acid used in the hydrothermal synthesis of NaYF_4_:Yb/Er provides sufficient hydrophilicity and biocompatibility of UCNPs without affecting their luminescence efficiency and cell labeling capacity [17,18]. A similar synthesis approach is used in the present study for obtaining Y_1−x_Gd_x_F_3_:Yb/Er UCNPs with a biocompatible surface. Er^3+^ is the most commonly used lanthanide ion for LIR-based thermometry in the physiologically relevant range of temperatures due to the outstanding thermal coupling between ^2^H_11/2_ and ^4^S_3/2_ energy levels, which is separated by ca. 750 cm^−^^1^. While the maximal thermal sensitivity (S) of ~1%K^−^^1^ is determined for YF_3_:Yb/Er UCNPs at 293 K [19,20], there is no data evidencing the thermal sensing capacity of GdF_3_:Yb/Er UCNPs. It is shown that along with NIR bio-imaging and enhanced photodynamic therapy [21], gadolinium-based UCNPs additionally provide a positive contrast effect in T1-weighted magnetic resonance imaging. Accordingly, they could be used as dual modality imaging agent biolabels [22]. Hence, the LIR-based temperature sensitivity and potential cytotoxicity of both, GdF_3_:Yb/Er and YF_3_:Yb/Er particles synthetized via chitosan assisted solvothermal synthesis, were investigated in this study. 

## 2. Materials and Methods

### 2.1. Materials 

All reagents were purchased from Sigma–Aldrich (St. Louis, MO United States) and used as obtained. Rare earth (RE) nitrates [Y(NO_3_)_3_ × 6H_2_O, Gd(NO_3_)_3_ × 6H_2_O, Yb(NO_3_)_3_ × 5H_2_O and Er(NO_3_)_3_ × 5H_2_O], sodium fluoride (NaF, ≥99%), anhydrous ethylene glycol (C_2_H_6_O_2_, 99.8%) and chitosan (CS, low molecular weight, 50,000–190,000 Da) were used for Y_0.8−x_Gd_x_F_3_:Yb_0.18_Er_0.02_ particles synthesis (x = 0, 0.15 and 0.8). In addition to the variation in the gadolinium content, two concentrations of the precursor solution were used for the synthesis of Gd_0.8_F_3_:Yb/Er, low (C_RE_^3+^: 2.5 mmol, CS: 50 mg) and high (C_RE_^3+^: 5 mmol, CS: 100 mg), so samples are marked as shown in Table 1.

### 2.2. Synthesis of Y_1−x_Gd_x_F_3_:Yb,Er Particles 

Up-converting particles with a nominal compositions Y_0.8_F_3_:Yb_0.18_Er_0.02_, Y_0.65_Gd_0.15_F_3_:Yb_0.18_Er_0.02_ and Gd_0.8_F_3_:Yb_0.18_Er_0.02_ were solvothermally synthetized in the presence of chitosan using the following precursor: RE-nitrates were dissolved in 10 mL of deionized water and mixed with 15mL of the chitosan solution; then, 10 mL of the NaF solution (F^+^/RE^3+^ = 7) and 35 mL of ethylene glycol were added; the obtained precursor was stirred for 20 min. The synthesis was performed with constant stirring (100 rpm) in a sealed PTFE linen autoclave at 200 °C for 2 h. After cooling, the product of the reaction was separated through centrifugation (8000 rpm), rinsed with ethanol and water several times, and dried at 90 °C (2 h). 

### 2.3. Characterization 

The phase composition of the samples was examined using X-ray powder diffraction (XRPD) at room temperature, using a Rigaku SmartLab diffractometer (Rigaku Europe, Neu-Isenburg, Germany) with CuKα radiation and the 2θ scanning rate of 0.01°/s. The structural data for the powders were acquired through LeBail and Rietveld refinement in Topas 4.2 (Bruker AXS GmbH, Karlsruhe, Germany) [23]. The morphology, particle size, and chemical composition were examined using a scanning electron microscope (SEM, Philips XL 30/EDAX-Dx4, Philips, Amsterdam, Netherlands) equipped with an energy dispersive X-ray detector (EDS). Transmission electron microscopy (TEM) combined with the selected area electron diffraction (SAED) study was performed on a JEOL JEM 2100 instrument (JEOL, Akishima, Tokyo, Japan) operated at an accelerating voltage of 200 kV. Prior to this, the samples had been ultrasonically dispersed in acetone and deposited on a holey carbon copper grid. DigitalMicrograph™ (DM, Gatan Inc., 3.7.4, Pleasanton, CA, USA) software was used for phase identification based on the fast Fourier transform (FFT) calculation of an image. The SEM images were used to analyze the particle size distribution. For every sample, the dimensions of at least 50 particles chosen randomly were measured. Fourier transform infrared spectroscopy (FTIR) was performed using a Nicolet iS10 FT-IR Spectrometer (Thermo Scientific Instruments, San Diego, CA, USA) in the spectral range from 400 to 4000 cm^−1^. Photoluminescence (PL) emission measurements were performed at room temperature using a TE-cooled CCD fluorescence spectrometer (Glacier X, BWTEK, Plainsboro, NJ, USA) and a 976 nm laser diode (single mode pigtailed BL976-SAG300 laser diode of 976 nm, Thorlabs, Newton, NJ, USA). For temperature-dependent measurements of PL intensity, a Peltier element (20 × 20 mm) was used along with the PID temperature controller (Wavelength electronics WTC32ND-EV, Wavelength electronics Inc., Bozeman, MT, USA) in the temperature range from 25 to 50 °C. The temperature of the upper surface of the element, on which the sample was placed, was measured using a 10 kΩ thermistor, bonded to the surface. The set temperature was maintained with the stability of 0.002 °C. In order to avoid the local heating of the sample, the excitation was performed via laser radiation in very short pulses of 10 ms, with frequency of 1 Hz, giving a fill factor of 1:100. The luminescent radiation was collected using a multimode optical fiber with a large diameter (0.6 mm) and a large numerical aperture (0.22), and further introduced into the spectrum analyzer. All spectrograms were recorded without pulse averaging and were fitted with 5 separate Gaussian spectral lines, and appropriate integrals were used to find the LIR.

### 2.4. Cell Lines

Human cells used for the testing of cytotoxic effects were ATCC cell lines MRC-5 (ECACC 84101801) and A549 (ATCC CCL-185). MRC-5 cells are normal human lung fibroblasts, used as a general model in routine testing for cytotoxicity, even for testing medical devices, as recommended by ISO 10993-5:2009(E) [24]. The A549 cell line is a line of cancerous lung epithelial cells, chosen as a model for testing the influence on lung epithelial cells, and also as a supplementary model for assessing the effect of materials on different cell types. The cells of both lines were maintained in Dulbecco’s Modified Eagle Medium with L-glutamine, supplemented with 10% of fetal bovine serum and 100 U/mL of the penicillin/streptomycin mix, in a humidified atmosphere at 37 °C and 5% CO_2_. The cell medium was changed every 2 days. Cells were passaged before reaching 80% confluency, using trypsinization with 0.1% trypsin/EDTA. 

### 2.5. Cell Viability Assay

For assessing the cytotoxic effect of the samples, an MTT colorimetric assay was performed. Cells (2 × 10^4^ cells/well) were seeded in 96 well plates, followed by incubation at 37 °C and 5% CO_2_ for 24 h. The medium was then discarded, and cells were exposed to three different concentrations (10, 25 and 50 µg/mL) of the samples. Prior to addition to the wells, the test samples were dispersed in the cell media, followed by vortex (3 × 1 min) and ultrasonic dispersion for 3 min, to obtain suspensions. After 24 h of incubation under the same conditions, the treatment medium was removed, and cells were carefully washed using 1x phosphate buffered saline. FA fresh medium with 0.5 mg/mL of MTT dye (3-[4,5-dimethylthiazol-2-yl]-2,5-diphenyltetrazolium bromide) was then added and plates were incubated for another 3 h, allowing for the formation of formazan crystals. After the removal of the medium containing MTT, crystals were dissolved using DMSO and by shaking the plate. Absorbance was recorded at 570 nm using a Multiskan plate reader (Thermo Scientific Instruments, San Diego, CA, USA) and average survival was calculated comparing the absorbance levels in treated cells with absorbance of non-treated, control groups. 

### 2.6. Cell Imaging by Laser Scanning Microscopy

For the visualization of GF-L uptake by A549 cells 50 μg mL^−1^ of sterile suspension was filtered through 0.45 μm syringe filter to separate agglomerates that could provoke saturation during imaging. Coverslips were cut into 8 × 8 mm square pieces and sterilized by using ethanol and UV light. Then, they were placed at the bottom of 12 well plates. Cells were trypsinized from flasks in which they were grown and seeded to the wells at the density of 105 cells/mL. After seeding, cells were incubated for 24 h at 37 °C and 5% CO_2_ to allow attachment and proliferation. The medium was then replaced with the fresh medium containing 50 μg/mL of GF-L nanoparticles, and plates incubated for another 24 h with the treatment, at the same conditions. On the third day, cells were gently washed for three times by using pre-warmed 1 × PBS, in order to wash out the remaining unbonded mesocrystals. An amount of 4% paraformaldehyde was applied for 20 min to allow the fixation of the cells. Then it was thoroughly washed with 1xPBS several times. Coverslips containing fixated cells were removed from the wells and placed with the cell-covered side facing down to the 20 μL drops of Mowiol on the microscopic slides. Samples were then left to dry on the ambient temperature for 24 h before they were observed under laser scanning microscopy. 

Two-photon excited (auto)fluorescence of A549 cells and the up-conversion of GF-L mesocrystals were recorded using a custom made nonlinear laser scanning microscope with the excitation wavelengths of 730 nm and 976 nm, respectively. Microscope set-up description is given elsewhere [17,18]. The signal was collected in back reflection, using an oil immersion objective lens with high a numerical aperture (EC Plan-NEOFLUAR, NA = 1.3; Carl Zeiss AG, Oberkochen, Baden-Württemberg, Germany). A visible wide range bandpass filter (400–700 nm) was used to filter out the laser and transmit only the signal. Obtained raw data was processed and analyzed using ImageJ software (1.47v, National Institutes of Health, Bethesda, MD, USA).

### 2.7. Assessment of the Cell Morphology Parameters and Fluorescence

Cell morphology in culture was inspected via bright field microscopy before and after the addition of treatment and during the incubation time. From the images obtained using the laser scanning microscope, cell morphology parameters were assessed as follows, cell size, in terms of cell diameter, surface and nucleus/cytoplasmic ratio. Nuclear circularity index was also calculated, as described in [25], using the Equation (1):

(1) $$Circularity=\frac{4\pi \times area}{{\left(perimeter\right)}^{2}}$$

To roughly estimate the amount GF-L mesocrystals association with different cells, average fluorescence per cell was assessed by measuring the pixel intensity from the laser microscopy images of MRC-5 and A549 with filtered fluorescence originating by GF-L mesocrystals. All measurements were performed by using ImageJ software.

### 2.8. Statistical Analysis

All cell viability tests were performed in triplicate, and in three independent experiments. The results of the viability of the treated cells were compared to the average absorbance of untreated control cells, and are presented in diagrams as the average values of the percent of survival, +/− standard deviation. Experimental and control groups were compared depending on the number of samples via Student’s *t*-test or one-way analysis of variance (ANOVA), followed by Tukey’s post hoc test, with *p* value being set to *p* < 0.05.

## 3. Results

**Figure 1:**
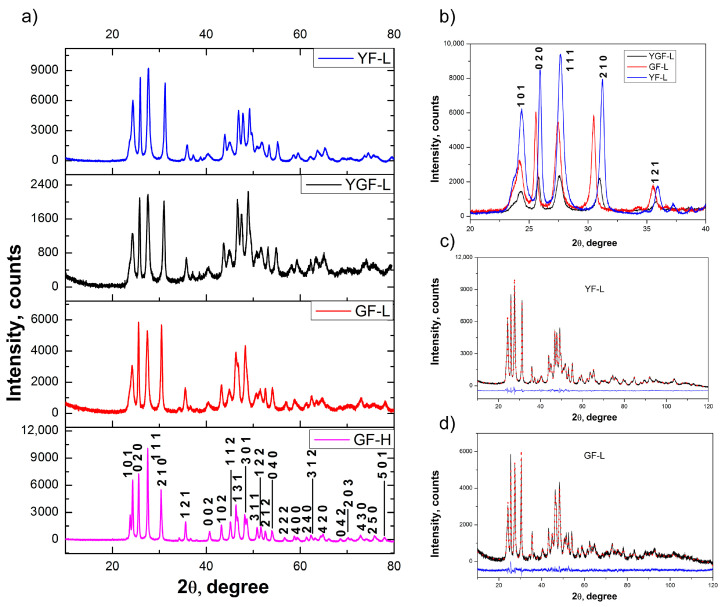
XRPD patterns of Y_0.8−x_Gd_x_F_3_:Yb_0.18_Er_0.02_ samples (**a**) reflection shifting due to Gd^3+^ content increase (**b**) and Rietveld refinement of samples YF-L and GF-L (**c**,**d**). In (**b**–**d**) the experimental data are shown as the black solid line while the red dotted pattern corresponds to the calculated data. The differences between the observed and calculated intensities are plotted in the blue line.

The XRPD patterns, presented at Figure 1a. indicate that all samples crystallize in the β-YF_3_ type orthorhombic crystal structure (S.G. *Pnma*), JCPDS 01-074-0911. In this structure, the Y^3+^ ion is coordinated by nine F^‾^ ions. Its substitution by Gd^3+^, which has a larger ionic radius (Gd^3+^:1.107 Å, Y^3+^:1.075 Å), led to an increase in crystal lattice parameters, due to which, the shifting of the (020), (111), (210) and (121) reflections towards lower values of 2Ɵ is notable, Figure 1b. The XRPD structural refinement (Figure 1c,d, Table 2), reveals the coexistence of two different particle populations in all samples (two phases of the same composition were used to adjust the experimental pattern), both adopting the same *Pnma* space group with similar unit cell parameters and a quite different crystallite size and strain. The smallest difference in the crystallite sizes of two populations was detected in the GdF-L sample, where both values were below 100 nm, Table 2. 

**Figure 2:**
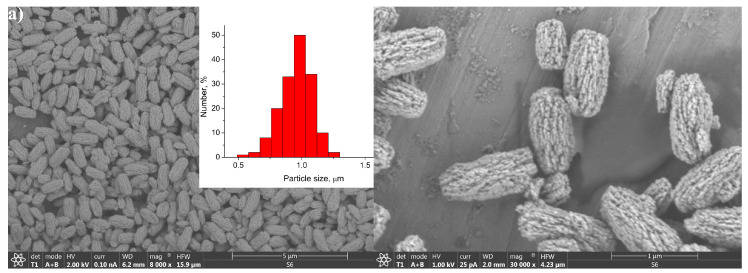

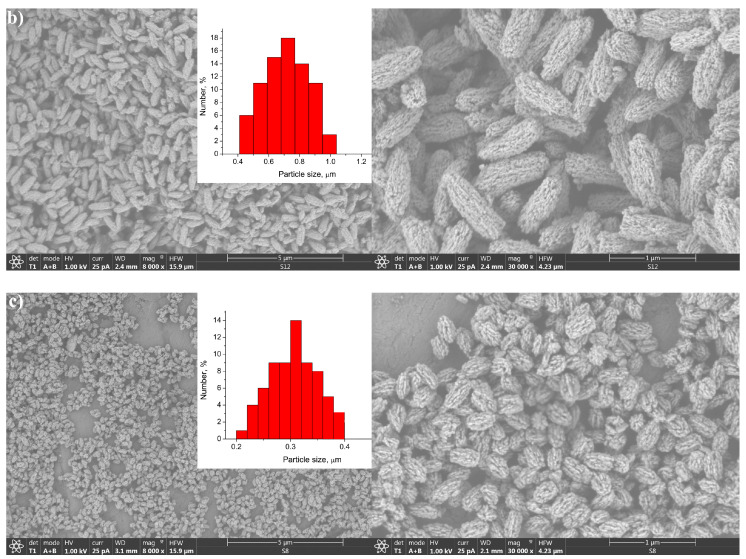
SEM of the samples YF-L (**a**), YGF-L (**b**), and GF-L (**c**), with the corresponding particle size distribution**.**

**Figure 3:**
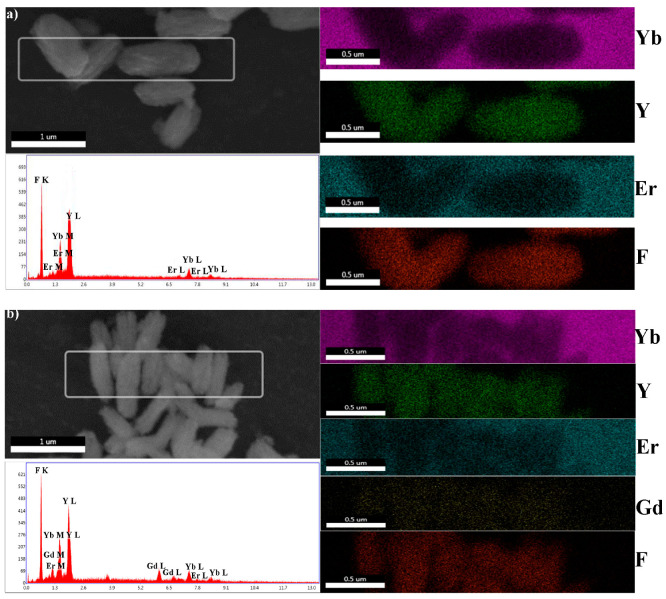

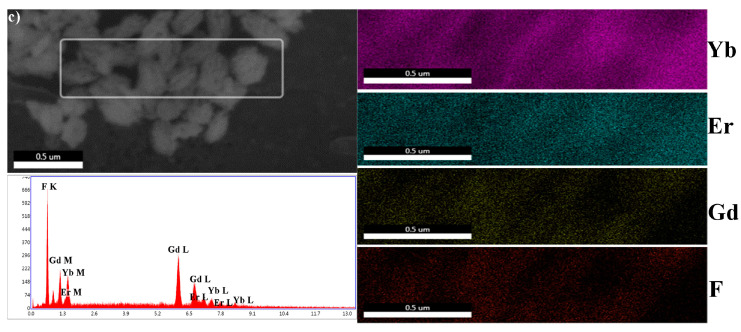
EDS analyses of the samples YF-L (**a**), YGF-L (**b**), and GF-L (**c**)**.**

The size and the morphology of the synthetized particles were revealed by (SEM and TEM. The particles obtained from the precursors with a low concentration have an elongated shape similar to unshelled peanuts, the length of which decreases from 1 µm to 300 nm as the gadolinium content increases, Figure 2. The particles appear to be non-agglomerated and quite uniform in size. As observed in Figure 2, they are composed of smaller nanocrystals and are porous to a certain degree. Their high purity and the uniform distribution of all constituting elements (yttrium: Kα at 14.931 and Lα at 1.92 keV; ytterbium: Lα at 7.414 and Mα at 1.404 keV; erbium: Lα at 6.949 and Mα at 1.521 keV; gadolinium; Kα at 6.053 and Mα at 1.181 keV; and fluorine: Kα at 0.677), are confirmed by EDS elemental mapping, Figure 3.

**Figure 4:**
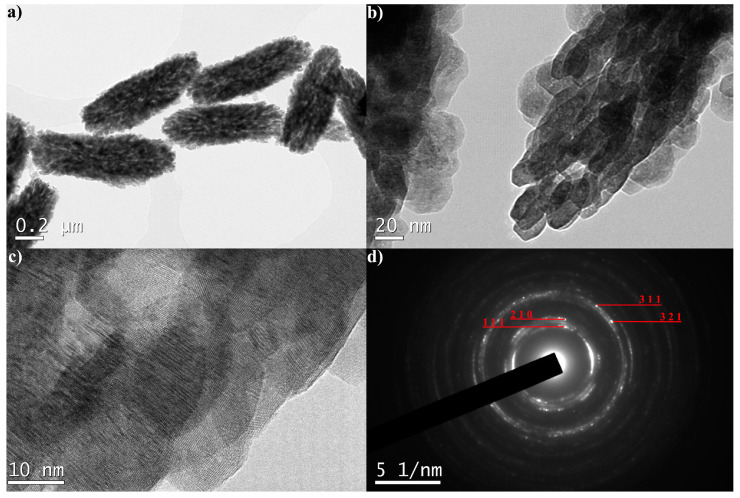
TEM images (**a**–**c**) and SAED pattern of YF-L mesocrystals (**d**).

**Figure 5:**
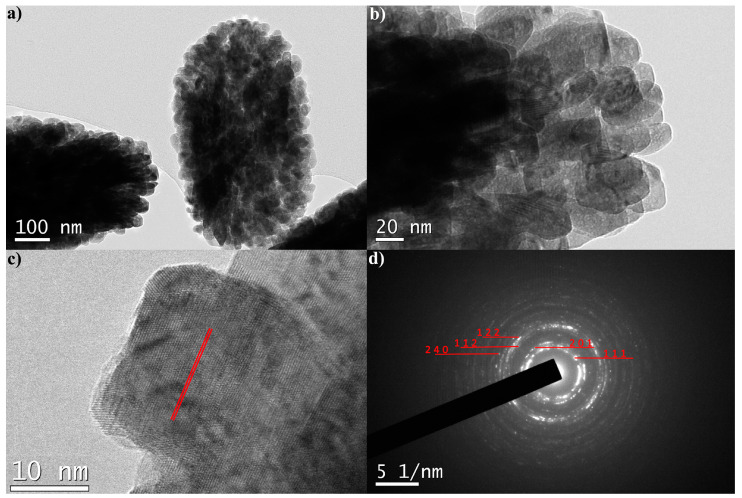
TEM images (**a**–**c**) and SAED pattern of GF-L mesocrystals (**d**). Red lines in (**c**) indicate crystal planes.

The TEM analysis of the YF-L and Gd-L samples confirms that the unshelled peanuts -shaped particles are mesocrystals formed by much smaller monocrystals, Figure 4 and Figure 5. The shape of these monocrystals is slightly elongated in the YF-L particles. As evident in Figure 4b, they are self-assembled to form rice-shaped mesocrystals by attaching to each other along the longer edge. The development of moiré fringes, visible in Figure 4c, suggests the internal displacement of crystal planes in elongated nanocrystals due to the lattice defects. The SAED pattern, Figure 4d, shows indexed diffraction rings of (111), (210), (311) and (321) crystal planes with d values of 3.20 Å, 2.85 Å, 1.80 Å and 1.60 Å, respectively. All values align well with corresponding ones from the JCPDS 01-074-0911 card. The GdF-L mesocrystals are composed of smaller quasi-spherical nanocrystals. Figure 5c, reveals the presence of (111) crystallographic planes of gadolinium fluoride. In addition, the monophase composition of particles is identified by the presence of the (111), (201), (112), (122) and (240) planes with d values corresponding well to those obtained through the XRPD analysis.

**Figure 6:**
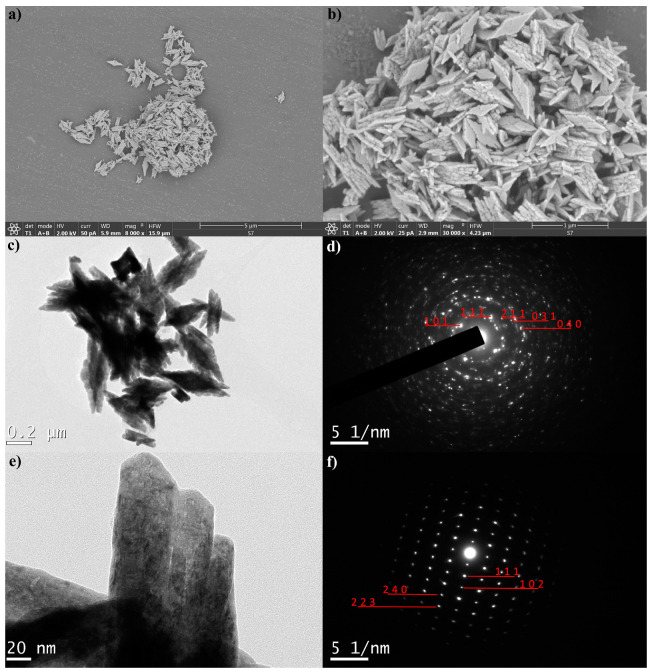
SEM (**a**,**b**) and TEM (**c**,**e**) and SAED images (**d**,**f**) of GF-H sample**.**

With the increased precursor concentration, a change in the particles’ morphology was detected. The formation of rhombic mesocrystals with a layered structure, and the diagonal length up to 0.4 µm, were revealed by the SEM analysis, Figure 6a,b. They are built by orientational ordering and layering of thin plates, Figure 6c. As it is notable from the TEM image shown in Figure 6e, the formation of plates is effectively guided by the oriented attachment of rod-like Gd_0.8_F_3_:Yb_0.18_Er_0.02_ monocrystals whose indexed diffraction spots are given in Figure 6f.

**Figure 7:**
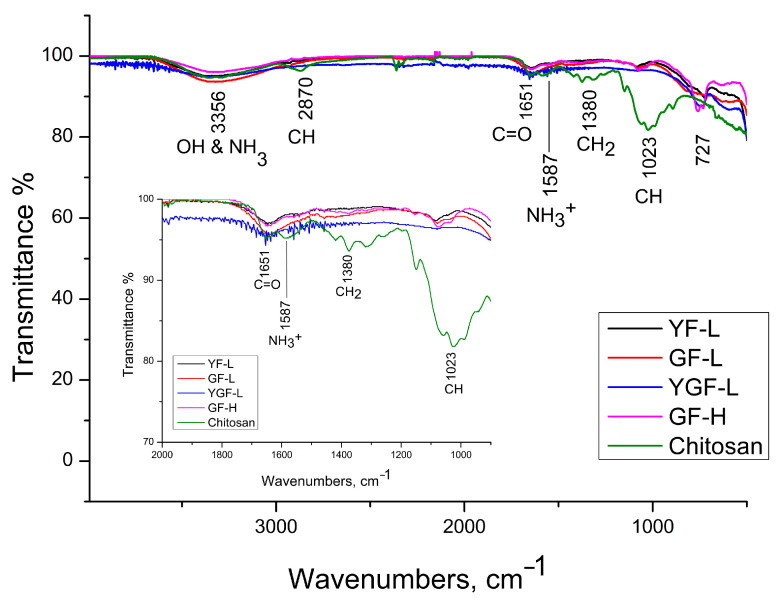
FTIR spectrum of samples and pure chitosan**.**

The Fourier transform infrared (FTIR) spectra of the synthetized samples and pure chitosan presented in Figure 7, indicate the preservation of chitosan ligands onto the surface of mesocrystals. According to the literature data [26,27,28], the spectrum of pure chitosan can be classified as follows: broad band at 3500–3000 cm^−^^1^ (stretching vibration of hydroxyl O-H which overlaps with amine N-H group); band at 2870 cm^−^^1^ (C-H bond in –CH_3_); bands at 1651.7 cm^−^^1^ and 1587 cm^−^^1^ (C=O stretching (amide I) and NH stretching (amide II), respectively); bands at 1417.9 cm^−^^1^ and 1374.1 cm^−^^1^(CH_3_ bending vibrations); band at 1149.9 cm^−^^1^ (asymmetric vibration of the CO group); a band near 1060.2 cm^−^^1^ (CO bending vibrations of the pyranose ring). The FTIR spectrum of the samples showed a decrease in adsorption and a slight shifting of chitosan related bands at: 3399.9 cm^−1^ (-OH and amine -NH group stretching), 1651.7 cm^−1^ (C=O stretching), 1557 cm^−1^ (protonated amine stretching), 1373.5 cm^−1^ (CH_2_ bending) and 1080.4 cm^−1^ (CO bending), implying its preservation at the particles surface.

**Figure 8:**
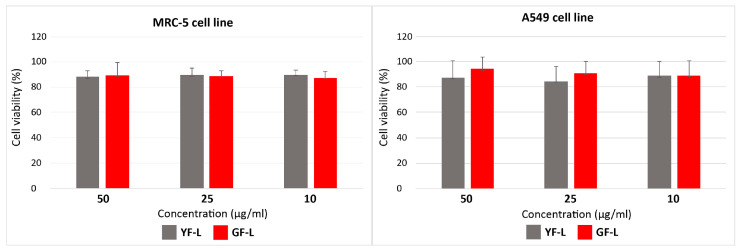
Cytotoxicity assay of YF-L and GF-L samples in MRC-5 and A549 after a 24-h exposure**.**

The viability of the MRC-5 and A549 cells exposed to the concentrations of 10, 25 and 50 µg/mL of YF-L and GF-L samples for 24 h, expressed as percentages of the control cells viability, are shown in Figure 8. As observed from the graphs, all cells treated exhibited more than 80% viability, at all concentrations of both samples. The cell viability was highly preserved after a 24-h exposure, being above 80% for all examined concentrations of both samples. There was a negligible difference between the groups treated with various concentrations of GF-L, where no concentration dependency was observed. On the other hand, the viability of A549 treated with YF-L at the concentration of 25 µg/mL, was found to be slightly reduced compared to the viability of MRC-5 cells (at the same concentration). These results confirm that both samples are outstandingly biocompatible with human cells.

**Figure 9:**
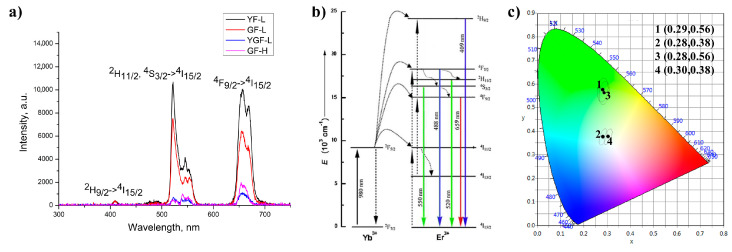
Up-conversion spectrum of the samples excited at 976 nm (**a**), energy level diagram of theYb^3+^/Er^3+^couple with radiative transitions marked by solid vertical blue, green and red lines (**b**), CIE diagram in which 1: YF-L; 2: YGF-L; 3: GF-L; and 4: GF-H (**c**).

Figure 9a shows the UC emission spectra of all samples under 976 nm excitation. The visible emissions from ^2^H_9/2_ → ^4^I_15/2_, ^4^F_7/2_ →^4^I_15/2_, ^2^H_11/2_,^4^S_3/2_ → ^4^I_15/2_, and ^4^F_9/2_ →^4^I_15/2_, transitions of Er^3+^ ions were detected at 409, 488, 520/550, and 659 nm, respectively. As shown in Figure 9b, the population of the excited states in Er^3+^ ions are due to energy transfer from the Yb^3+^ sensitizer. Initially, Yb^3+^ ions were excited from the ^2^F_7/2_ ground state to the ^2^F_5/2_ excited state through the absorption of the incident photons. This energy is transferred further to the ^4^I_11/2_ state of the Er^3+^ ion. The population of the ^4^I_11/2_ state also occurs through the direct excitation of Er^3+^ ions from their ground ^4^I_15/2_ state. The population of the higher ^4^F_7/2_ and ^4^F_9/2_ Er^3+^ levels occurs either through energy transfer from another excited Er^3+^ ion which is in close proximity, or through a two-step energy transfer from Yb^3+^ to the neighboring Er^3+^ ions. The populated ^4^F_7/2_ state of Er^3+^ ions relax radiatively to the ground ^4^I_15/2_ state or non-radiatively decay to the ^2^H_11/2_ and ^4^S_3/2_ states, from which radiative de-excitations to the ground ^4^I_15/2_ state generate green emission at 520 nm (^2^H_11/2_→ ^4^I_15/2_) and 550 nm (^4^S_3/2_ → ^4^I_15/2_). Red emission appears due to the ^4^F_9/2_ → ^4^I_15/2_ de-excitation which could be additionally intensified by the non-radiative ^4^F_7/2_ → ^4^F_9/2_ relaxation. The strengthening of the red emission in YGF-L and Gd-H samples could be a consequence of the appearance of an additional mechanism which involves a successive energy transfer by three photons excitation, as it is corroborated in our previous work [29] and also in [30], but further studies are needed to confirm it. Weak blue emission at 409 nm is due to radiative relaxation of the highest ^2^H_9/2_ state, which is populated by successive energy transfer [29,31,32,33]. Since the intensity of each emission is determined by the probability of radiative and non-radiative transitions in particles, as well as their crystallinity, the calculated CIE color coordinates differ, as it is shown in Figure 9c. 

Evidently, the luminescence of YF-L and GF-L samples is much stronger than that of the YGF-L and GF-H ones. For these samples, temperature sensing capability was determined in the physiologically relevant range of temperatures from 25 °C to 50 °C, using the LIR method. The LIR method for temperature measurement utilizes the intensity ratio of emissions from a pair of closely spaced upper levels, that can be considered as thermally coupled (TCL) [34]. TCL means that these levels have such a small energy difference that the higher level is thermally populated from the lower, so that their relative population follows a Boltzmann distribution. The LIR of spectral lines arising from the transitions from the TCLs level to a common terminal level is [35]:

(2) $$LIR(T)=\frac{I_{H}(T)}{I_{L}(T)}=B\cdot e^{-\frac{{∆E}_{HL}}{k\cdot T}}$$

where I_H,L_ are the luminescence intensities from the upper (H) and lower (L) TCLs to the terminal level radiative transition; ΔE_HL_—the energy difference between these two levels; k—the Boltzmann constant; and T—the absolute temperature. B is the temperature invariant parameter depending on the host material. 

The potential of a material for temperature measurement applications is quantified through the absolute and relative change in the LIR with temperature. The absolute sensitivity of LIR to temperature is given by the partial derivative:

(3) $$S_{a}=\left|\frac{\partial LIR}{\partial T}\right|=\frac{{∆E}_{HL}}{kT^{2}}\cdot B\cdot e^{-\frac{{∆E}_{HL}}{k\cdot T}}$$

The relative sensitivity is given by:

(4) $$S_{r}=\frac{S_{a}}{LIR}=\frac{{∆E}_{HL}}{kT^{2}}\cdot 100\%$$

The accuracy and precision of an LIR-based temperature measurement system depend on the sensitivities given above, and also on many characteristics of the measurement system, among which the signal/noise ratio in the captured spectrogram is the most important. However, all characteristics that cause measurement uncertainty can be jointly quantified by the standard deviation of the measured LIRs, obtained over many measurements, taken at a certain fixed temperature. If we denote this quantity by σ_a_, the precision of the temperature measurement ΔT can be calculated by the expression:

(5) $$∆T=\frac{\sigma_{a}}{S_{a}}$$

The accuracy of the measurement is, to the great extent, equal to the precision ΔT defined above, but it also includes additional non-measurable parameters, such as the accuracy of setting the real sample temperature and a possible error due to local heating, which both we considered negligible. 

**Figure 10:**
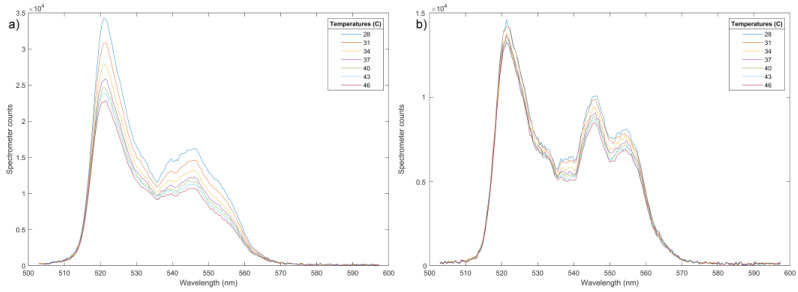
Temperature-dependent UC emission spectra of the YF-L (**a**) and GF-L (**b**) in the wavelength range of 500–600 nm**.**

**Figure 11:**
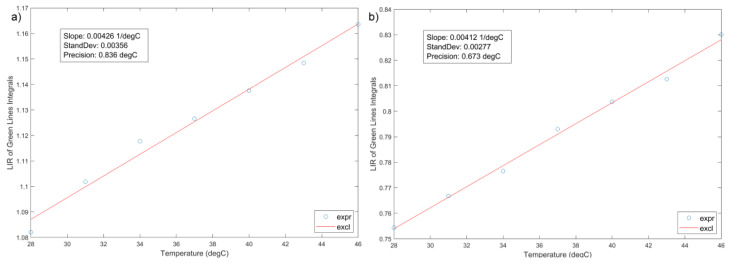
LIR values of YF-L (**a**) and GF-L (**b**) as a function of temperature (dots) and the corresponding line obtained by linear fitting**.**

The green parts of spectrograms recorded for YF-L and GF-L in the physiologically interesting temperature range of 25 °C to 50 °C, are shown in Figure 10a,b. A clear decrease in the intensity of individual lines with increasing temperature is clearly notable. The difference in the change rate in the intensity of individual lines is not visible to the naked eye because of the relatively narrow temperature range. In the Yb-Er^3+^ type systems, TCL levels which are of interest are usually ^4^S_3/2_ and ^2^H_11/2_ levels of Er^3+^, and their transitions to the level ^4^I_15/2_, are taken to calculate the LIR. These transitions are traditionally labeled as 550 nm and 520 nm in Figure 9b, although they have slightly different values, as can be seen in Figure 10. Also, it is evident that there are more than these two lines in the luminescence spectra shown in Figure 10, and that line intensities cannot simply be read as the local maxima of the spectrograms. Therefore, more realistic values of the centers and widths of these lines were obtained by deconvoluting of the recorded spectrograms, using the fitting that assumes a Gaussian shape of the lines. The initial values for the deconvolution process, performed using MATLAB, were determined from the spectrograms as the positions and widths of the obvious peaks. The resulting LIRs for different temperatures are shown in Figure 11a,b.

The obtained experimental data can be fitted using Equation (2), although in such a narrow temperature range, the linear approximation is quite correct. The slope of both linear curves is equal to Sa = 0.0042 °C^−^^1^, which can be considered as the absolute sensitivity for temperature measurement in the temperature interval of interest. The relative sensitivity has, according to Equation (4), strong temperature dependance, but it can also be approximated with a constant value in the interval of interest, which is equal to S_r_ ≈ Sa/LIR_310_, where LIR_310_ is the LIR in the middle of the interval, see Figure 11. The relative sensitivities are therefore 0.38%/°C and 0.53%/°C, for YF-L and GF-L samples, respectively. These values are smaller than those obtained using (4) when the energy differences ΔE_HL_ of the levels whose centers are read as local maxima of the spectrogram in Figure 10 are used. This phenomenon is described in [36], while later in [37] is shown that it occurs, to a greater or lesser extent, in most of the investigated materials. It is mainly caused by non-radiative energy transitions, overlapping the TCL spectra, and luminescence originating from other energy levels. 

The measurement uncertainty in this work was determined as the standard deviation of LIR measurement, taken separately for both samples on the set of results obtained in 32 consecutive measurements at the temperature of 37 °C. These quantities in principle depend on temperature, but in our measurements, we consider the value obtained at 37 °C as a valid one over the relatively narrow temperature range. The calculated values of the uncertainty for the YF-L and GF-L samples are σ_YF_ = 0.0036 and σ_GF_ = 0.0028, respectively. 

The precisions of measurement ΔT_YF_ of 0.84 °C and ΔT_GF_ of 0.67 °C for the YF-L and GF-L samples, respectively, are calculated using Equation (5). These values are slightly higher than those reported in other studies of similar systems [8], which is due to the lower relative sensitivity, discussed in the text above, and the higher noise in the spectrogram originating from the recording method, based on a single laser pulse of 10 ms, without averaging.

**Figure 12:**
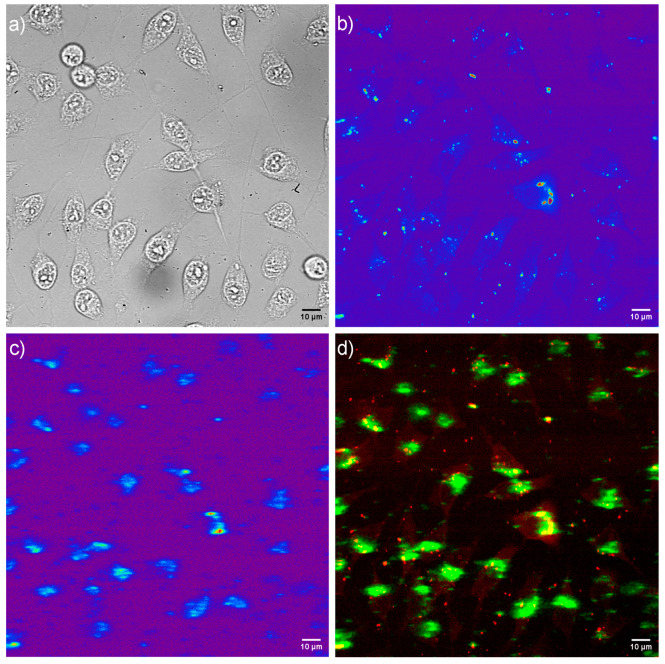
Laser scanning microscopy images of MRC-5 cells incubated with 50 μg mL^−1^ of GF-L mesocrystals; bright field image of cells (**a**), cells auto-fluorescence upon femto-second excitation at 730 nm (**b**), image of GF-L mesocrystals upon continuous wave excitation at 976 nm (**c**), and their positioning in cells, revealed through co-localization of the cell auto-fluorescence and the GF-L photoluminescence (**d**)**.**

**Figure 13:**
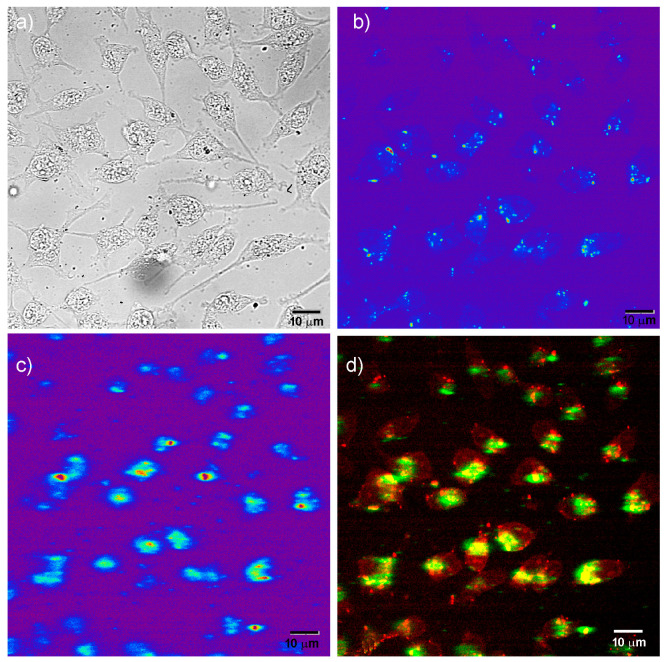
Laser scanning microscopy images of A549 cells incubated with 50 μg mL^−1^ of GF-L mesocrystals; bright field image of cells (**a**), cells auto-fluorescence upon femto-second excitation at 730 nm (**b**), image of GF-L mesocrystals upon continuous wave excitation at 976 nm (**c**), and their positioning in cells, revealed through co-localization of the cell auto-fluorescence and the GF-L photoluminescence (**d**).

**Figure 14:**
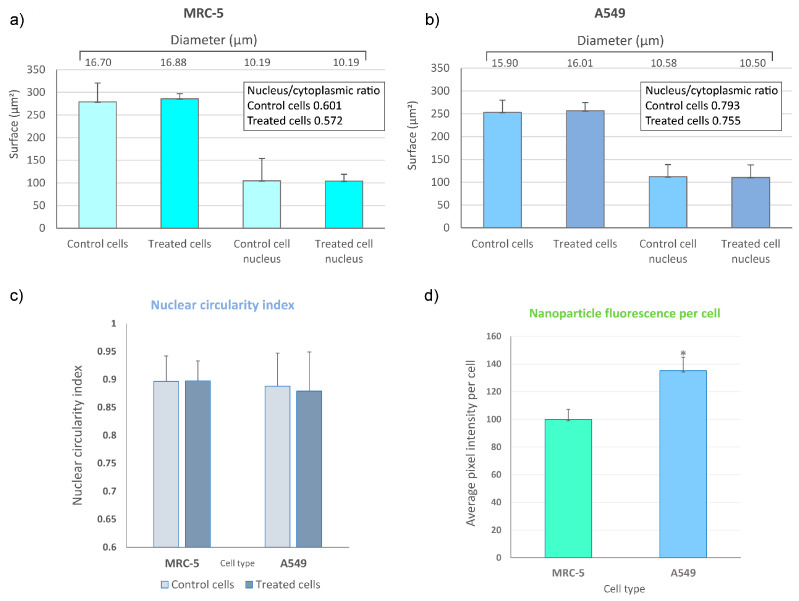
Comparison of cell morphology parameters in terms of average surface, diameter and (in inserts) nucleus/cytoplasmic ratio in control and GF-L-treated group of MRC-5 (**a**) and A549 cells (**b**); nuclear circularity indexes of control and treated cells (**c**); average fluorescence per cell, as calculated from the pixel intensity in filtered images. Measurements were performed by using laser microscopy images and ImageJ software. Statistical significance threshold was *p* < 0.05 and is represented by *(**d**).

To monitor the intracellular uptake and non-specific cell labelling in vitro, GF-L unshelled peanuts-shaped particles were incubated with MRC-5 and A549 cells. Laser scanning microscopy images are shown in Figure 12 and Figure 13. The bright field images of the cells shown in Figure 12a and Figure 13a, reveal that a characteristic fibroblast-like shape was preserved in all cells incubated with GF-L, so they maintained same growth pattern as non-treated control cells. A pseudo color images of the cells auto-fluorescence upon femto-second excitation at 730 nm are shown in Figure 12b and Figure 13b, whilst the pseudo color images of GF-L mesocrystals upon excitation at 976 nm are given in Figure 12c and Figure 13c. Overlapping the images marked with “b” and “c” in Figure 12 and Figure 13 implies that GF-L mesocrystals (visible as green fluorescence spots) are positioned in the cells cytoplasmic area adjacent to the plasma membrane, in both cell lines. Such positioning, without disturbing cells nuclei confirms successful cell labeling and enables the sensing of the temperature in cells. Laser scanning microscopy images were used further to assess the cell morphology parameters and “occupation degree” of cells by mesocrystals (based on the fluorescence). Obtained results are presented in Figure 14. In both cell types, none of the tested morphological parameters exhibited statistically significant change following the treatment with the GF-L mesocrystals. Cell diameter, surface and nucleus/cytoplasmic ratio remained of the approximately the same values as in non-treated cells. Nucleus circularity index was also unchanged. As regarding fluorescence levels in MRC-5 and A549, normal cells had around 26% lower pixel intensity (fluorescence) per cell as compared to cancer cells. However, despite this statistically noteworthy result, the difference is not compelling enough to provide selectivity towards cancer cells labeling.

## 4. Discussion

According to the phase diagram of NaF-YF_3_ and YF_3_-GdF_3_ systems [38], the crystallization of the following fluorides could be achieved in function of temperature, NaYF_4_ in two forms (cubic α phase, *Fm-3m*; and hexagonal β phase, *P63/m*), and three crystal structures of the Y_1−x_Gd_x_F_3_ phases (orthorhombic β phase, *Pnma*; hexagonal, *P63cm*; and trigonal, *P-3c1* phase). Contrary to our previous work, where we obtained the β-NaYF_4_:Yb,Er phase under identical processing conditions [39], since the YF_3_ and GdF_3_ phases show complete miscibility, all samples synthesized in this study, independent of stoichiometry, have the same orthorhombic crystal structure. The XRPD analysis revealed a change in the unit cell parameters due to the substitution of ytterbium by gadolinium and the coexistence of two different particle populations in all samples, which is associated with the formation of monocrystals.

Considerable progress has so far been made in the synthesis of YF_3_ particles with different morphologies, such as truncated octahedral-, quadrilateral-, hexagonal-, spherical-, octahedral-, and bundle-like nanocrystals [40]. We also reported the change in the morphology of the YF_3_ monocrystal during EDTA-assisted hydrothermal synthesis—provoked by the modification of the processing parameters [29], as well as the compositional change which occurred in the course of sonication [41]. In this study, the change in the particle morphology was detected with the rise of the Gd concentration. In the GF-L sample, a decreased size of particles (compared to the YF-L and YGF-L samples) could be associated with the higher charge density on their crystal surface, which slows the diffusion of negatively charged fluoride ions leading to a reduction in the crystal growth rate [42]. With the rise of the gadolinium precursor concentration, a change in the morphology is detected. A high tendency towards self-organization of elongated Gd_0.8_F_3_:Yb_0.18_Er_0.02_ monocrystals into two-dimensional rhomboidal layers is probably due to the anchoring of branched chitosan ligands, which are present in a higher content in the GF-H sample. 

The existence of chitosan ligands at the particle surface ensures that mesocrystals have a low cytotoxicity. A low cytotoxicity of chitosan-capped YF_3_:Yb,Er nanoparticles toward human breast cancer MCF-7 cells and GdF_3_:Yb^3+^/Er^3+^/Li^+^ hollow spheres, reported in the literature [43,44], is in agreement with the results obtained in this study. The viability of both, MRC-5 and A549 cells was highly preserved after a 24-h exposure, being above 80% for all examined concentrations of YF-L and GF-L particles. As demonstrated in our previous study [17], the presence of chitosan amino functional groups at the αNaYF_4_:Yb,Er UCNPs surface enhances their use for in vitro cell labelling. The same feature is confirmed here for GF-L unshelled peanuts-shaped particles. Their successful incubation in the cytoplasmic region of MRC-5 and A549 cells enables the visualization of cells, and also creates condition for in situ measuring of cell temperature. Morphological parameters of the cell, and especially nucleus, are considered to be indicators of the change in cellular physiological condition. As it is shown here, GF-L mesocrystals did not induce significant change in cell size parameters, excluding large-scale processes such as early-stage necrosis (necrotic volume increase) [45]. Homeostasis of nucleus/cytoplasmic ratio, indicative for many cellular physiologic activities, changes in growth and also pathological processes, was also unchanged [46]. Furthermore, the nucleus circularity index showed that there was also no change in the nucleus morphology. Nuclear circularity index values close to 1 indicate a normal, circular nucleus [25]. All of these parameters in the range of normal cell values did not exclude possibility of mesocrystals affecting the structures and physiology of the cells, but they did show that no morphology- and physiology-changing effects took place. This, together with the results from viability testing using MTT, is overall a good indication of biocompatibility of tested samples at this stage of research. This is of crucial importance, because mesocrystals obtained in this study have potential for application in temperature sensing in the physiologically relevant range of temperatures. 

The absolute sensitivity of 0.0042 °C^−^^1^, and the accuracy of 0.84 °C (YF-L) and 0.67 °C (GF-L) is somewhat lower than the previously published ones related to strongly agglomerated and irregularly shaped Y_0.78_Yb_0.2_Er_0.02_F_3_ particles obtained through solid-state reactions [19] and from glass ceramics embedded with YF_3_:Yb,Tm,Er nanocrystals synthesized using a melt-quenching method [20]. Both were determined for the much wider temperature range, in which the population of Er^3+ 2^H_11/2_ and ^4^S_3/2_ thermally coupled levels is governed by the Boltzmann distribution, and is not comparable with the thermal sensitivity of the GF_3_:Yb,Er phase due to the absence of published data.

## 5. Conclusions

Biocompatible Y_0.8−x_Gd_x_F_3_:Yb/Er mesocrystals with an unshelled peanuts-like, and rhombic-layered structure were obtained using chitosan-assisted solvothermal processing. These structures are built from much smaller monocrystals, whose tendency towards self-organization is governed by the quantity of preserved chitosan ligands at their surface. The difference in charge density inside the crystals, induced by the increase in the gadolinium content in the YF_3_ host matrix, resulted in a minor change in local symmetry of dopant ions, due to which the shape and intensity of Er^3+^ emission lines varied. The change in the visible green emission intensity with temperature, originating from the thermalization of the ^2^H_11/2_ and ^4^S_3/2-4_ levels of Er^3+^, is used for testing their capacity for temperature sensing. The detected absolute sensitivity of 0.0042 °C^−^^1^, the accuracies of 0.84 °C (Y_0.8_Yb_0.18_Er_0.02_F_3_) and 0.67 °C (Gd_0.8_Yb_0.18_Er_0.02_F_3_), in vitro cell labeling capacity, and the low cytotoxicity to both, MRC-5 and A549 cells, make them suitable for further optimization towards more specific application in biomedicine.

## Figures and Tables

**Table 1:** Composition of samples and precursors.

Nominal Composition	Sample Name	C_RE_^3+^ (mmol)	CS (mg)
Y_0.8_F_3_:Yb_0.18_Er_0.02_	YF-L	2.5	50
Y_0.65_Gd_0.15_F_3_:Yb_0.18_Er_0.02_	YGF-L	2.5	50
Gd_0.8_F_3_:Yb_0.18_Er_0.02_	GF-L	2.5	50
Gd_0.8_F_3_:Yb_0.18_Er_0.02_	GF-H	5	100

**Table 2:** Refined microstructural parameters of Y_1−x_Gd_x_F_3_:Yb/Er samples.

Sample	Unit Cell Parameters (Å)			CS (nm)	Strain	R_Bragg_
	a	b	c			
YF-L	6.2926 (3)	6.8621 (3)	4.4984 (3)	207 (23)	0.323 (5)	1.1
	6.3098 (5)	6.8605 (5)	4.4400 (5)	18 (1)	0.130 (1)	1.3
YGF-L	6.3331 (5)	6.8749 (5)	4.4453 (5)	20 (1)	0.120 (1)	1.6
	6.3239 (5)	6.8713 (5)	4.5050 (2)	183 (10)	0.280 (2)	2.2
GF-L	6.4685 (4)	6.9586 (4)	4.4604 (4)	23 (1)	0.174 (6)	1.1
	6.4590 (4)	6.9565 (4)	4.4011 (4)	64 (2)	-	2.5
GF-H	6.4751 (4)	6.9490 (3)	4.4256 (8)	120 (6)	0.195 (3)	1.4
	6.4885 (2)	6.9602 (2)	4.4318 (1)	37 (1)	-	1.7

## Data Availability

Data are contained within the article.
